# An improved preparation procedure for pollen samples from coastal clastic sediments

**DOI:** 10.1016/j.mex.2023.102016

**Published:** 2023-01-13

**Authors:** Qiang Yao, Kam-biu Liu, Erika Rodrigues

**Affiliations:** a Department of Oceanography and Coastal Sciences and Coastal Studies Institute, Louisiana State University, Baton Rouge, LA 70803, USA; b Institute of Geosciences, University of São Paulo, São Paulo, Brazil

**Keywords:** Palynology, Microfossil, Pollen, Palynomorph, Paleoecology, microfossil pollen preparation

## Abstract

Palynological analysis is a time-tested analytical method in the field of geology, geography, and archaeology across the globe. However, a major problem in palynology is that due to the dynamic nature of coastal and lake settings, sediment samples from these environments usually contain large proportion of clastic materials that are difficult to remove and isolate from pollen grains. In this study, we present a step-by-step procedure of an optimized preparation method to eliminate the organic and clastic materials and concentrate the pollen grains. We also present some insights on how to prepare a clean microscopic slide with intact pollen grains. We believe this procedure can successfully eliminate organic and clastic materials and concentrate the pollen grains to produce an ideal microscopic slide for pollen analysis.

• Extract samples and eliminate carbonate materials with hydrochloric acid.
• Remove organic materials with potassium hydroxide.
• Remove clastic materials with sieving and hydrofluoric acid.

## Graphical abstract

**Figure:**
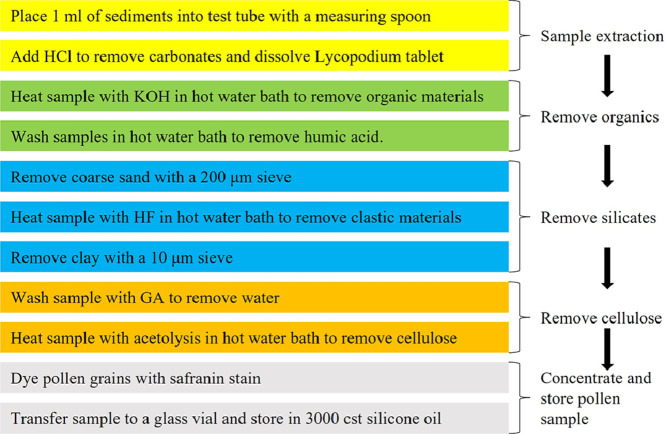

Specifications table

**Table:** 

Subject area:	Earth and Planetary Sciences
More specific subject area:	Palynology
Name of your method:	microfossil pollen preparation
Name and reference of original method:	N/A
Resource availability:	N/A

## Method details

### Rationale

Pollen analysis (palynology) is a time-tested analytical method in the field of geology, geography, and archaeology. It is widely used in the reconstruction of paleoenvironmental change and vegetation dynamics on timescales ranging from decades to millions of years [1]. On a shorter timescale, pollen analysis can be utilized to study natural disturbances such as storms [2], floods [3], and tsunamis [4]. On a longer timescale, palynology has been successfully used to document the patterns of sea-level fluctuations [5], monsoon dynamics [6], and climate changes [7,8]. Thus, the results of pollen analysis can provide a unique perspective for geologists and ecologists.

However, certain environmental settings such as the coastal zone are not only highly dynamic and complex, but also frequently encounter natural and anthropogenic disturbances. In particular, because of its labor-intensive nature of this analytical procedure, several factors can significantly affect the accuracy and efficiency of microscopic pollen analysis. One major problem is that due to the dynamic nature of coastal and lake settings, sediment samples from these environments usually contain large portions of clastic materials that are difficult to remove and isolate from pollen grains. A poorly prepared pollen sample will prolong the labor hours, but the usage of various chemicals may cause deformation and misidentification of the pollen grains. Thus, a proper preparation procedure should produce clean microscopic slides with intact pollen grains. In this study, we present a step-by-step procedure of an optimized preparation method and some insights on how to eliminate the organic and clastic materials and concentrate the pollen grains.

## Required materials and instruments

### General materials

1. Nitrile Exam Gloves
2. Weigh boat
3. 1 ml measuring spoon
4. 200 µm nylon or metal mesh sieve
5. 10 µm nylon or metal mesh sieve
6. 15 ml Nalgene test tube (acid, alkaline, and heat resistant)
7. Eye goggle
8. Stirrer
9. Measuring cylinder
10. Laboratory flasks

### Chemical materials

1. Deionized water
2. Hydrofluoric acid
3. Hydrochloric acid (10%)
4. Potassium hydroxide (10%)
5. Glacial acetic acid
6. Acetic anhydride
7. Sulfuric acid
8. Tertiary butyl alcohol
9. Silicone oil (3000 cst)

### Essential instruments

1. Binocular light microscope (Fig. 1a)

**Fig. 1:**
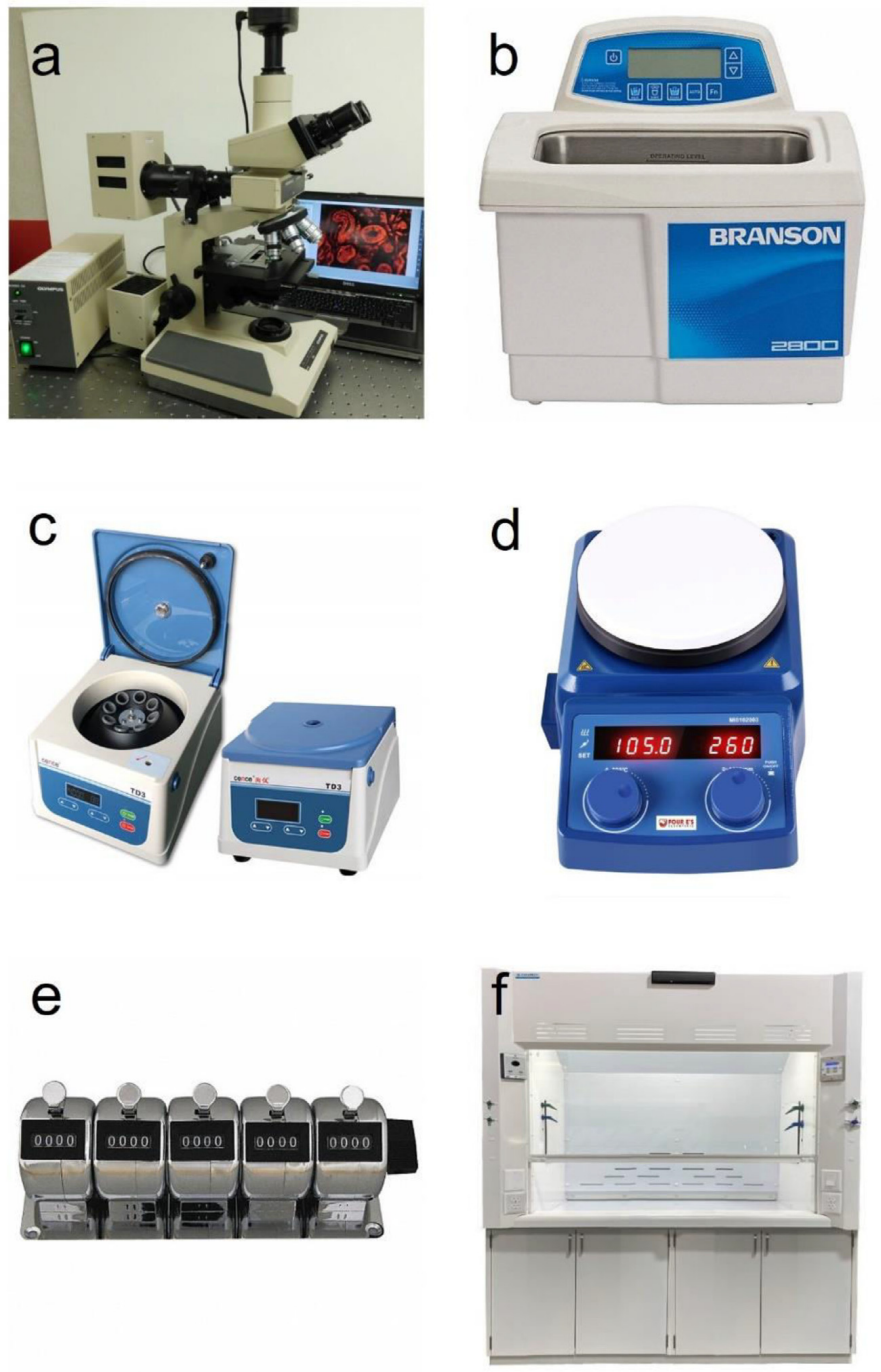
Essential instruments for microfossil pollen preparation, such as (a) binocular light microscope, (b) sonicator, (c) centrifuge, (d) magnetic hotplate stirrer, (e) tally counter, and (f) laboratory fume hood.
2. Sonicator (Fig. 1b)
3. Centrifuge (3500 RPM; Fig. 1c)
4. Magnetic hotplate stirrer (Fig. 1d)
5. Tally counter (Fig. 1e)
6. Acid-resistant fume hood (HF-grade) (Fig. 1f)

## Procedures (all the steps should be performed inside a fume hood)

### Sample extraction and elimination of carbonates

1. Sample the sediment core at designated intervals. Scrap the surface clean or take material from inside the core where contamination is least likely. Pack sediment into 1 ml measuring spoon and transfer to numbered Nalgene test tube.
2. Add 5 ml of HCl (10%) to each test tube and stir thoroughly. Add 1 Lycopodium tablet (exotic marker) to each test tube. Allow tablets to dissolve, then add another 5 ml of HCl to fill the test tube. The HCl will dissolve any carbonates in the sample as well as the exotic marker. Some fizzing is normal. Soak the sediments in HCl overnight to thoroughly breakdown the carbonate content.
3. Centrifuge and decant on the next day. Record each step in the laboratory notebook.

### Elimination of humic acid and organic materials with potassium hydroxide

4. Prepare “hot water bath” by heating 350 ml of water in a 500 ml beaker to boiling point on the hotplate. Add water as necessary to keep the water level adequate throughout the processing.
5. Wash and decant - fill test tube with DI water and stir sediments thoroughly, then centrifuge and decant.
6. Add ∼10 ml of KOH (10%) to each test tube, stir well, and heat in the hot water bath for 5 min. KOH deflocculates the sample, breaks down organic molecules, and removes humic compounds. The liquid in the test tube will appear dark brown if the sediments are highly organic. Centrifuge and decant.
7. Wash with water, heat in hot water bath for 2–5 min, centrifuge, and decant. This step accelerates the decomposition of humic compounds and lowers the pH of the solution. This step should be repeated until the supernatant is clear. If supernatant is still dark after 3 washes, step 6 and 7 should be repeated.

### Elimination of clastic materials with sieving and hydrofluoric acid

8. Sieve each sample with the 200 µm sieve and collect the fine fraction (<200 µm) of the sieved sediments. Since most pollen grains are smaller than 200 µm in size, this step eliminates the clastic material (i.e., sand) with diameter larger than 200 µm.
9. Add ∼10 ml HF (hydrofluoric acid) to each test tube, stir thoroughly, and heat in hot water bath for ∼5 min. Centrifuge and decant. HF dissolves silicates (i.e., silt and diatom). This step eliminates the clastic materials that passed through the 200 µm sieve.
10. If some fine grain silicate is still present in the bottom of the test tube, sieve each sample with the 10 µm sieve inside the sonicator and collect the coarse fraction (>10 µm) of the sieved sediments. Since most pollen grains are larger than 10 µm in diameter, this step eliminates the fine grain clastic materials (i.e., clay) with a diameter less than 10 µm.
11. Wash and decant.

### Elimination of organic matter and cellulose with acetolysis solution

12. Wash with GA (glacial acetic acid). Centrifuge and decant. This step acidifies the sediments and eliminates water in preparation for acetolysis, which will be violent if water is present.
13. Prepare the acetolysis solution in a glass graduated cylinder. Use 9 parts acetic anhydride to 1 part concentrated sulfuric acid. This protocol processes 6 samples at once, therefore, pour 54 ml of acetic anhydride into the cylinder. Then add 6 ml of sulfuric acid, drop by drop, to the acetic anhydride. Allow sufficient time for the acid to sink to the bottom of the cylinder and mix. If acid is added too rapidly, a violent reaction will occur.
14. Add ∼10 ml acetolysis solution to each test tube, stir well, and heat in boiling water bath for ∼2 min. Acetolysis dissolves cellulose. Thus, liquid in test tube should start to turn brownish. Centrifuge and decant carefully. The acetolysis solution reacts with water in the sink; thus, pour it out steadily but slowly.
15. Wash with GA, centrifuge, and decant.
16. Wash with DI water, centrifuge, and decant.

### Concentrate and store the pollen samples

17. Stir the sediments, add 1 drop of safranin stain, mix immediately with vortex mixer, and then fill test tube with TBA (tertiary butyl alcohol), stir thoroughly, centrifuge, and decant. Note that TBA can freeze at room temperature. If this occurs, place the TBA bottle in a hot water bath to thaw.
18. Transfer the finished pollen sample from test tube to labeled glass vial by first adding a few drops of TBA in the test tube, then stir it well using a vortex mixer, and decant into the glass vial. Repeat this step 2–3 times until all sediment from the test tube is transferred to the glass vial. Centrifuge the glass vials.
19. Add ∼1 ml of silicone oil in the glass vial, stir well, and record. Leave the vials uncapped overnight to evaporate the excess TBA.
20. On the following day, stir samples well to prevent clumping, add ∼1 ml silicone oil, label, and cap vials. Labels should include location, core number, sample number (or depth level), and processing date. Secure label with scotch tape.

## Method validation

**Fig. 2:**
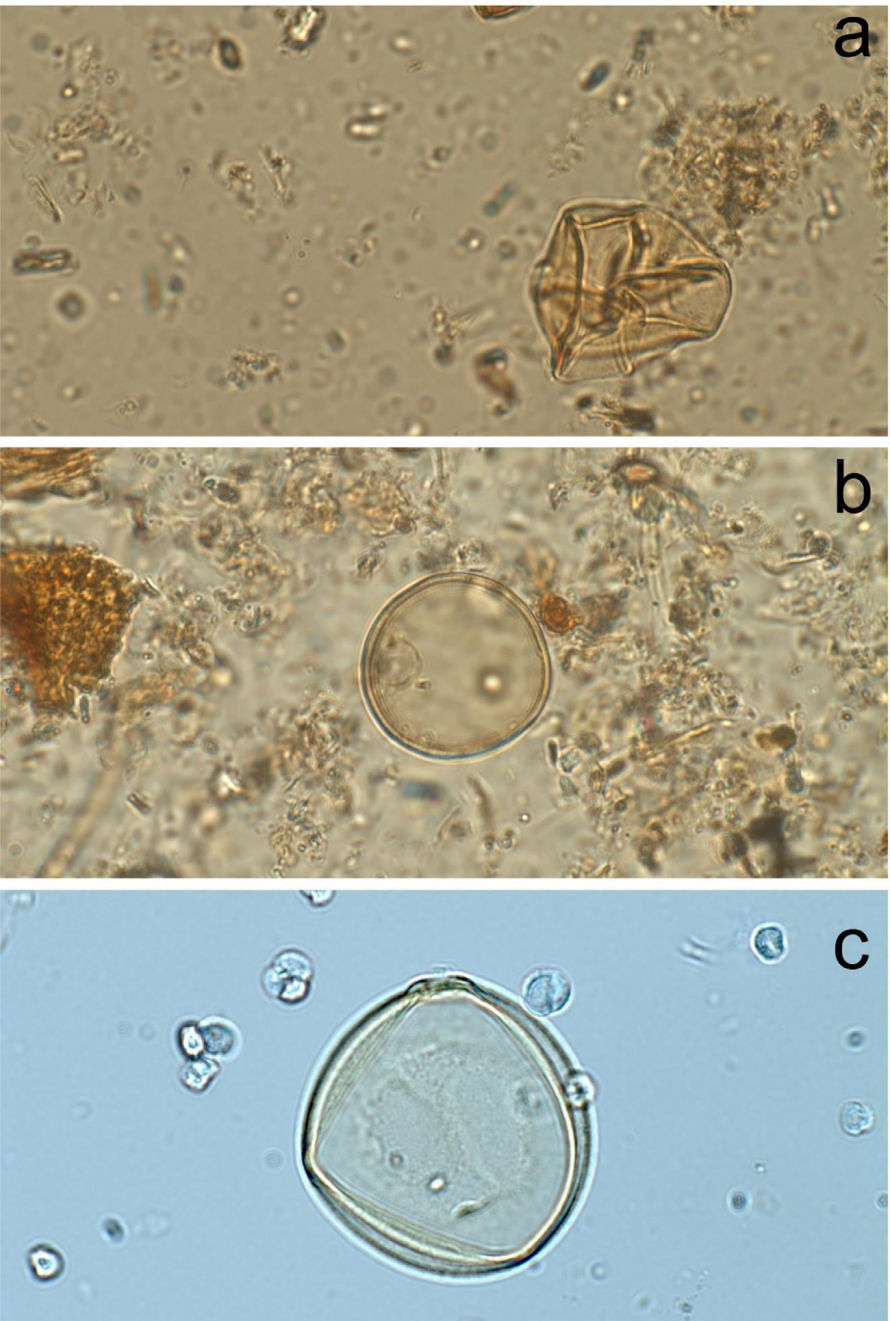
A comparison of light micrographs of Poaceae pollen grain following (a) this procedure but the sample was heated with HF in hot water bath for 10 min (double the recommended time); (b) this procedure without sieving and HF; and (c) this procedure.

In general, this procedure can remove the majority of the clastic sediments, organic matter, and cellulose, while preserving the shape and integrity of the pollen grains. Among all the steps, we consider sieving and hydrofluoric acid being the most critical step to remove clastic materials. However, hydrofluoric acid can still disintegrate certain pollen taxa with thin exine, such as Poaceae and Cyperaceae (Fig. 2a). A controlled experiment comparing the Poaceae pollen grains show that Poaceae pollen is the most intact when hydrofluoric acid is omitted during the processing procedure (Fig. 2b&c). However, in some cases clastic materials cannot be removed completely, and the pollen sample may still be difficult to identify (Fig. 2b). Thus, operators should use hydrofluoric acid judiciously based on the composition of the sediment sample. A general guideline will be to avoid using hydrofluoric acid when most clastic materials can be removed with sieving (Fig. 2c).

During the past five years, we have processed over 500 microfossil pollen samples using this preparation procedure and published a total of 13 palynological research articles in SCI journals [3], [4], [5], [6], [7], [8], [9], [10], [11], [12], [13], [14], [15]. If unpublished data are included, we have processed over 800 microfossil pollen samples using this preparation procedure. Thus, we believe this procedure can successfully eliminate organic and clastic materials and concentrate the pollen grains to produce an ideal microscopic slide for pollen analysis.

## Conclusion

In this study, we present an optimized procedure that can remove organic and clastic materials and increase accuracy for microfossil pollen identification. We want to point out that: (1) Each step should be recorded in the laboratory notebook during the pollen processing procedure; (2) Pollen sample should be stirred thoroughly during each step to ensure a complete reaction with the chemicals; (3) All test tubes need to be filled to the same level to ensure a good balancing in the centrifuge; (4) Water must be running continuously inside the fume hood drain to flush out all the chemicals used during pollen processing; and (5) Processing can be suspended overnight after steps 3,7,11 and 16.

## Ethics statements

This study does not involve any human subjects or animal experiments.

## Related research article

Yao, Q., Liu, K.B., Rodrigues, E., Fan, D. and Cohen, M., 2022. A palynological record of mangrove biogeography, coastal geomorphological change, and prehistoric human activities from Cedar Keys, Florida, USA. Science of The Total Environment, p.160189, https://doi.org/10.1016/j.scitotenv.2022.160189.

## CRediT authorship contribution statement

**Qiang Yao:** Methodology, Writing – original draft. **Kam-biu Liu:** Supervision. **Erika Rodrigues:** Data curation.

## Declaration of Competing Interest

The authors declare that they have no known competing financial interests or personal relationships that could have appeared to influence the work reported in this paper.

## Data Availability

No data was used for the research described in the article.
